# The Application of Single-Cell RNA Sequencing in Mammalian Meiosis Studies

**DOI:** 10.3389/fcell.2021.673642

**Published:** 2021-08-18

**Authors:** Yiheng Peng, Huanyu Qiao

**Affiliations:** Department of Comparative Biosciences, University of Illinois at Urbana-Champaign, Urbana, IL, United States

**Keywords:** meiosis, single-cell RNA-seq, transcription, transcriptome, spermatocyte, oocyte, spermatogenesis, oogenesis

## Abstract

Meiosis is a cellular division process that produces gametes for sexual reproduction. Disruption of complex events throughout meiosis, such as synapsis and homologous recombination, can lead to infertility and aneuploidy. To reveal the molecular mechanisms of these events, transcriptome studies of specific substages must be conducted. However, conventional methods, such as bulk RNA-seq and RT-qPCR, are not able to detect the transcriptional variations effectively and precisely, especially for identifying cell types and stages with subtle differences. In recent years, mammalian meiotic transcriptomes have been intensively studied at the single-cell level by using single-cell RNA-seq (scRNA-seq) approaches, especially through two widely used platforms, Smart-seq2 and Drop-seq. The scRNA-seq protocols along with their downstream analysis enable researchers to accurately identify cell heterogeneities and investigate meiotic transcriptomes at a higher resolution. In this review, we compared bulk RNA-seq and scRNA-seq to show the advantages of the scRNA-seq in meiosis studies; meanwhile, we also pointed out the challenges and limitations of the scRNA-seq. We listed recent findings from mammalian meiosis (male and female) studies where scRNA-seq applied. Next, we summarized the scRNA-seq analysis methods and the meiotic marker genes from spermatocytes and oocytes. Specifically, we emphasized the different features of the two scRNA-seq protocols (Smart-seq2 and Drop-seq) in the context of meiosis studies and discussed their strengths and weaknesses in terms of different research purposes. Finally, we discussed the future applications of scRNA-seq in the meiosis field.

## Introduction

Meiosis has been studied for decades. Sexual reproduction requires meiosis to generate haploid gametes from diploid germline cells. Starting at early prophase I (leptonema), mammalian meiotic chromosomes condense gradually and pair to their homologous chromosomes (homologs) with the help of DNA double-strand breaks (DSBs). At the beginning of the next stage (zygonema), a protein structure called Synaptonemal Complex (SC) forms to link homologs at several chromosome sites, accompanied by exchanging genetic materials at the crossover sites that usually originate from the SC initiation sites (Page and Hawley, 2004). The SCs zip up all the homologs from end to end except sex chromosomes at the entry of pachynema and disassemble at late prophase I (diplonema). Homologs with exchanged genetic material are aligned at metaphase I and separated at anaphase I, followed by a second cell division in meiosis II to separate sister chromatids (Bolcun-Filas and Handel, 2018). The complex and highly organized events during meiosis require the strict and unique transcription regulation during meiosis. For example, a generally low transcription level was observed from leptonema to early pachynema, followed by a rapid increase to reach a transcriptional peak at late pachynema (Eddy and O’Brien, 1997; Margolin et al., 2014; da Cruz et al., 2016). However, the insight mechanisms of the transcription regulation remain unclear.

In mammals, germ cells develop in divergent ways in the two sexes: “spermatogenesis” in males and “oogenesis” in females. In males, spermatogenesis happens throughout the lifespan of most mammals after puberty, generating billions of gametes continuously (De Kretser et al., 1998). In females, however, oocyte pools form in the ovaries before birth with limited numbers, and the pools reduce sharply around birth and gradually throughout the female reproductive lifespan (Reynaud and Driancourt, 2000). Transcription regulations are also different between spermatogenesis and oogenesis. Spermatocytes have XY bodies that have an unique structure and transcription patterns during meiosis; however, the XY bodies are not found in oocytes due to lacking the Y chromosome (Handel, 2004). Besides, some meiosis-specific genes are regulated differently through spermatogenesis and oogenesis (Yang and Wang, 2018; Mihola et al., 2019). For instance, the mutations of *Spo11, Msh5*, and *Sycp3* arrest spermatocytes at zygotene stage, but allow oocytes to go further (Hunt and Hassold, 2002). All these sexual differences in meiosis require further studies to investigate unique sex-specific regulations.

Understanding the molecular mechanisms in meiosis is important for human reproductive health. Errors of meiotic events cause abnormalities in germ cells, leading to infertility, miscarriage, and genetic diseases. Infertility, for example, is a global issue of public health. Infertility affects 9–15% of the male population, and up to 18% of the female population (Practice Committee of the American Society for Reproductive Medicine, 2006; Barratt et al., 2017; Barbieri, 2019). Due to the variety of causes that lead to infertility, 5% of cases are diagnosed as “unexplained” (Unuane et al., 2011). To investigate those molecular mechanisms of diseases that induce reproductive diseases, especially to find biomarkers for clinic applications, canonical meiotic studies take advantages of proteomic techniques and cytological approaches, to capture unique features for diseases (DeSouza et al., 2005; Parmar et al., 2008; Chen et al., 2009). However, these studies only target limited proteins and genes, and fail to draw a comprehensive map of the transcription network, making it inefficient for finding new biomarkers.

Development of the next generation sequencing provides possibilities for nucleotide studies at a genome-wide level. Bulk RNA-seq, a sequencing-based approach, is a powerful tool to study meiotic molecular mechanisms due to its ability to fully sequence the whole transcriptome. In basic science, by using bulk RNA-seq, transcriptional profiles of germ cells have been studied (Margolin et al., 2014; Ball et al., 2016; Zhao et al., 2020c). Meiotic transcriptional profiles of different cell types generated by RNA-seq provide information on how the transcriptome is regulated at different stages, which further enable researchers to study transcriptionally regulated biological processes. In clinical utility, bulk RNA-seq has been developed as a powerful tool to discover biomarkers linked to various diseases, including reproductive diseases (Kumar et al., 2019; Liu et al., 2019; Zhang C. et al., 2019). However, meiosis is a long process and has multiple stages in both males and females, and bulk RNA-seq takes a large amount of cells as the input, and the average gene expressions of the input cells as the output. Thus, bulk RNA-seq fails to detect the heterogeneities among the input cells and is unable to study unknown cell types or rare cell populations, in both basic and clinical studies.

A more advanced RNA-seq method, single-cell RNA-seq (scRNA-seq), breaks the aforementioned limitations by improving the sequencing resolution to the single-cell level (Tang et al., 2009; Navin et al., 2011; Xu et al., 2012). Until now, more than twenty scRNA-seq protocols have been generated. To capture transcriptomic information from individual cells and build single-cell sequencing libraries, scRNA-seq methods include single-cell isolation and lysis, mRNA capture, cDNA generation by reverse transcription, and cDNA amplification (Kolodziejczyk et al., 2015; Hedlund and Deng, 2018). Unlike bulk RNA-seq, cells used by scRNA-seq have to be lysed separately so that mRNAs released from one cell can be separated from other cells. Depending on different scRNA-seq protocols, cDNA libraries of individual cells can be distinguished either by physical separation (multi-well plates) or by barcode sequence labeling (unique molecular identifiers [UMI]). Therefore, transcriptomic heterogeneity among individual cells can be detected. This allows scRNA-seq to capture minor cell groups with unique transcriptomic features that are harder to detect by bulk RNA-seq. scRNA-seq also can potentially capture more biomarkers specific for many cell types in clinical studies.

Here, in this review, we summarized recent discoveries in meiosis studies that applied scRNA-seq. We also discussed how different scRNA-seq approaches, together with their downstream analysis methods, contribute to mammalian meiosis-related studies.

## Why Should We Apply scRNA-Seq to Spermatogenesis and Oogenesis Studies?

### ScRNA-Seq Applications in Spermatogenesis Studies

In recent years, the spermatogenesis field has had some breakthroughs achieved by scRNA-seq. ScRNA-seq helps generate more comprehensive transcriptome profiles, discover new cell types and gene functions, and find certain cell type abundance in tissues.

I. ScRNA-seq generates comprehensive transcriptome profiles for mammalian spermatogenesis. Margolin et al. (2014) generated transcriptomes for mouse spermatogenesis by using bulk RNA-seq that failed to reveal cellular heterogeneity. Since 2017, several scRNA-seq studies have broken the limitations of the bulk RNA-seq (Guo et al., 2017, 2020; Li et al., 2017; Chen et al., 2018; Green et al., 2018; Hermann et al., 2018; Lukassen et al., 2018; Wang et al., 2018; Ernst et al., 2019; Grive et al., 2019; Wen and Tang, 2019; Shami et al., 2020). While bulk RNA-seq based transcriptome profiles can only distinguish the main meiotic stages, scRNA-seq studies further separated the known stages into finer substages. E.g., preleptotene cells (between mitosis and meiosis entry), usually have a small population, were able to be split into four substages: pre-meiotic/G1 phase preleptotene stage, early S phase preleptotene stage, middle S phase preleptotene stage, and late S phase preleptotene stage (Chen et al., 2018). On the other hand, bulk RNA-seq data can be combined with scRNA-seq results to facilitate staging spermatocytes. In one study, juvenile testis bulk RNA-seq, juvenile testis scRNA-seq, and adult testis scRNA-seq, were used together to precisely stage germ-cell development in mice (Ernst et al., 2019). Due to the semi-synchronization of the first wave of spermatogenesis, scRNA-seq data from each time point of the juvenile testis represented different stages through spermatogenesis, which was confirmed by histology. Their bulk RNA-seq data of juvenile testis further verify the cell-type classification results. This study generated a comprehensive transcriptome for spermatogonia differentiation and meiosis, especially for leptotene and zygotene spermatocytes in early prophase I (Ernst et al., 2019). However, as another scRNA-seq study pointed out that the transcriptional profile of the first wave is different from the subsequent spermatogenesis waves in mice (Grive et al., 2019), the scRNA-seq analysis of the first-wave spermatogenesis in juvenile testis may be problematic. Therefore, various factors should be taken into consideration for combining different approaches in scRNA-seq analysis. In summary, scRNA-seq provides high-resolution data for mapping germ-cell development in mammalian testes.
II. ScRNA-seq helps discover new cell types involved in spermatogenesis. ScRNA-seq data records the transcriptomic information for all cells. Thus, new cell types can be separated from other cell types by using clustering methods. The stage of the new cell types can be identified in cell progression trajectories (Details in section “ScRNA-seq downstream analysis for meiosis studies”). Guo et al. (2017) identified several transitional stages during spermatogonia-stem-cell differentiation in humans by scRNA-seq. Similar cell types were also defined by other three groups (Chen et al., 2018; Green et al., 2018; Tan et al., 2020), revealing transcriptional transitions between mitosis and meiosis. In general, scRNA-seq helps to dissect main meiotic stages into finer substages.
III. ScRNA-seq facilitates finding novel gene functions during spermatogenesis. ScRNA-seq allows gene enrichment analysis for investigating transcriptional dynamics between different cell groups. By comparing different cell groups, differentially expressed genes can be investigated, indicating their potential roles in a certain cell subtype. Specifically, gene upregulation and downregulation can be achieved by comparing subsequent stages. Chen et al. (2018) found the fibroblast growth factor (FGF) signaling pathway was repressed at the mitosis-to-meiosis transitions, suggesting the suppression of the FGF pathway may be required for entering meiosis. Wang et al. (2018) discovered a series of genes specifically expressed in certain spermatogenic stages. For instance, *Tdrg1*, *Ccdc112*, and *Aurka* can be used to distinguish zygotene, pachytene, and diplotene, respectively, although their meiosis-specific functions need to be further explored. By comparing the sequencing data between wild-type and mutant mice, scRNA-seq could also help understand the roles of certain genes in meiosis (Fang et al., 2019; Jung et al., 2019).
IV. ScRNA-seq can help dissect chromosome-wide transcriptional profiles during spermatogenesis. Similar to RNA-seq, the relative transcriptional level of cells can be calculated for studying chromosome transcriptional status. For instance, Lau et al. (2020) found that Meiotic Sex Chromosome Inactivation (MSCI) can be quantitively determined by calculating expression ratio between sex chromosomes and autosomes by scRNA-seq data analysis. Compared to conventional methods, scRNA-seq provides more detailed information on the duration and silencing patterns of MSCI. Besides, those genes escaped from the MSCI could also be identified.

### Comparing Meiotic scRNA-Seq Studies Between Male and Female

A lot of scRNA-seq approaches for spermatocytes can be used for oocyte studies as well. However, not all those protocols and analysis methods can be applied to oocytes due to its unique features. First, oocytes are less abundant than spermatocytes. Most of the oocyte-related scRNA-seq studies used only a few of oocytes, making it challenging to study oocyte heterogeneity. Second, unlike spermatogenesis, mammalian oogenesis is not a continuous process to generate clear trajectories. Primary oocytes arrest at dictyate stage for decades and resume upon stimulation of the luteinizing hormones. The ovulated oocytes are halted again at Metaphase II (MII) until fertilization (Esencan and Seli, 2018; Li et al., 2019). Third, oocytes are surrounded by granulosa cells, making large-scale cell isolation challenging. Because of the aforementioned reasons, most of the current scRNA-seq studies focus on oocytes at GV, MI, and MII stages with limited cell numbers in each experiment, which makes it hard to compare male and female germ-cell transcriptomes at single-cell level (Ferrero et al., 2019; Zhang T. et al., 2019; Ye et al., 2020; Yu et al., 2020; Yang et al., 2021).

It is not until recent years that scRNA-seq was applied to transcriptome studies of early prophase I oocytes (Li et al., 2017; Ge et al., 2020; Niu and Spradling, 2020; Wang et al., 2020). Li et.al. (2017) combined magnetic-activated cell sorting (MACS) and fluorescence activated cell sorting (FACS) to isolate male and female human fetal germ cells (FGCs) for scRNA-seq. Their results showed that female FGCs are in both somatic and meiotic stages, while all the male FGCs do not reach meiosis, indicating a non-synchronized manner for male and female FGC development. This non-synchronized manner makes it hard to directly compare male and female transcriptomes because of their different meiotic progression status. Meanwhile, their data can only separate female meiosis into three main stages: meiosis entry, meiotic prophase, and oogenesis. Wang et al. (2020) performed single-cell RNA-seq on monkey oocytes by collecting follicles from four ovaries and generate a transcriptome trajectory of the oocytes isolated from primordial to antral follicles. 418 oocytes were finally remained in the data and further separated to four subgroups of oocytes that were from primordial, primary, secondary, and antral follicles, respectively. Since this study focuses more on folliculogenesis rather than oogenesis, meiosis-related transcriptional transition was not fully discussed here. Recently, Niu and Spradling (2020) and Ge et al. (2020) independently performed scRNA-seq on a large number of mouse meiotic oocytes using 10X drop-seq platform, for the first time, revealing clear transcriptome profiles of early meiotic stages. Their data showed some shared early meiotic markers, such as *Stra8, Dusp9*, and *Rhox9*, and late meiotic markers, such as *Zcwpw1* and *Tex15*. However, due to different analysis approaches, a large portion of meiotic markers does not match with each other. To further compare meiotic transcriptomes between oocytes and spermatocytes, we compare Ge’s oocyte marker data with spermatocyte data from Jung et al. (2019). Although there are similarities between oocytes and spermatocytes, the most significant leptotene oocyte marker, *Actb*, is not dominant in spermatocyte leptonema. Instead, *Actb* is transcribed in undifferentiated spermatogonia and spermatids. In general, 10 out of 20 leptotene/zygotene oocyte markers are also found in leptotene/zygotene spermatocytes. Remaining genes are frequently shown in spermatogonia, indicating a transcriptional delay of oocytes at early prophase I compared to spermatocytes. Only 2 out of 10 most significant oocyte pachytene markers share with spermatocytes, while none of the dictyate oocyte top makers overlap with prophase spermatocyte markers. This analysis reveals a significant difference of the meiotic transcriptomes between oocytes and spermatocytes.

### The Application of scRNA-Seq in Meiosis-Related Diseases

ScRNA-seq is not only applied to basic research but also to clinical studies. Infertility, for example, is a common disease that threatens human health. The cause and diagnosis of infertility are complicated and diverse. Generally, causes of infertility can be categorized into three groups: female-related, male-related, and mixed. Until now, no causes have been identified for 10% of infertile couples (Zitzmann, 2013; Deroux et al., 2017; Krausz and Riera-Escamilla, 2018).

Meiotic defects can induce infertilities and many idiopathic infertility cases might be meiosis-related. Therefore, it is important to investigate the molecular mechanisms of how meiotic defects link with infertility (Hanson et al., 2017). However, most of the previous studies were conducted at the cell or tissue levels. The investigation of the diseases normally rely on cytology, which were not able to go further for deeper insight of their molecular mechanisms (Yatsenko and Rajkovic, 2019). To reveal the genetic causes of the idiopathic infertility, Next-Generation Sequencing (NGS) was developed as a clinical tool for finding genetic mutations by sequencing the entire genomes of patients (Oud et al., 2019). The functions of the candidate genes discovered by DNA-seq can be further studied by analysis of the knockout mice. However, gene-knockout studies normally focus on limited molecular pathways, but fail to cover the genome-wide transcriptional network. Due to these limitations, the studies failed to capture the insight mechanisms of the diseases, especially at the transcriptional level. Thus, novel tools are needed to study molecular mechanisms of the meiosis-related diseases, particularly at the genomic level.

In recent years, scRNA-seq has been developed as a powerful tool to identify new genes associated with female infertility. In females, due to its ability for genome-wide and single-cell level transcriptome profiling, scRNA-seq is used to identify new genes associated with infertility by tracking differentially expressed genes between normal and defective cells. In a recent case study, an infertile female patient had repeated multipronuclei (MPN) formed in her zygotes. To investigate gene expression profiles of oocytes and zygotes, scRNA-seq was performed with downstream gene ontology (GO) analysis to compare the patient’s and normal cells. Three candidate genes were identified based on their meiosis-related GO functions and their different expression patterns between patient and normal cells (Dai et al., 2017). This study demonstrated that scRNA-seq can be used to discover gene regulation alterations in disease studies. Another scRNA-seq study focused on recurrent total fertilization failure (RTFF) patients. Suo et al. (2018) compared transcriptional profiles between normal oocytes and abnormal oocytes like poly-pronuclei and pronuclear-stage-arrest oocytes. Several differentially expressed meiosis-related genes were found by Kyoto Encyclopedia of Genes and Genomes (KEGG) analysis of the scRNA-seq data (Suo et al., 2018). However, the validation of the roles for those genes in diseases requires more data collected from new clinical cases. Similarly, scRNA-seq was also used to study polycystic ovary syndrome (PCOS) and to evaluate the transcriptomic alteration caused by PCOS (Liu et al., 2016). In this paper, the authors collected oocytes and cumulus cells at GV, MI, and MII stages from patients and normal donors. After identification of differentially expressed genes by scRNA-seq analysis, KEGG analysis was performed to find potentially related pathways. Many genes, like *Ppp2r1a* and *Egfr*, were increasingly expressed in PCOS oocytes, which can help us find out the causes of PCOS (Liu et al., 2016). However, further scRNAseq and genetic studies are needed to verify these results.

ScRNA-seq has also been used as a novel tool for male infertility diagnosis. In a recent study, scRNA-seq was performed in testis samples from a non-obstructive azoospermia (NOA) patient. Normal testis samples have both somatic and germline cells. However, no germline cells were found in the testis of this patient by scRNA-seq, which is consistent with the histological staining results—spermatocyte depletion in the patient testis. Interestingly, many genes related to male reproduction were differentially expressed in the Sertoli cells of the NOA patient, which provides information for studying the mechanism of NOA-associated infertility (Wang et al., 2018).

Taken together, scRNA-seq is a powerful research tool for studying human male and female infertility. Specifically, this high-resolution sequencing method can be used to identify defective mutations by combining GO with KEGG analysis, to evaluate treatment efficiency, and to identify abnormal cell populations in patients.

## How Can We Conduct scRNA-Seq to Study Meiosis?

### What Sequencing Strategies to Choose for Meiosis Studies?

Choosing the appropriate sequencing strategy is the first and important step for scRNA-seq studies. Various scRNA-seq experimental approaches were used in previous meiosis studies, e.g., CEL-seq2, Drop-seq, MARS-seq, Smart-seq, and Smart-seq2 (Ramsköld et al., 2012; Picelli et al., 2013; Jaitin et al., 2014; Macosko et al., 2015; Hashimshony et al., 2016). They differ from each other by the methods of library generation and sequencing; consequently, they differ in sensitivity, cost, and sequencing depth.

**FIGURE 1:**
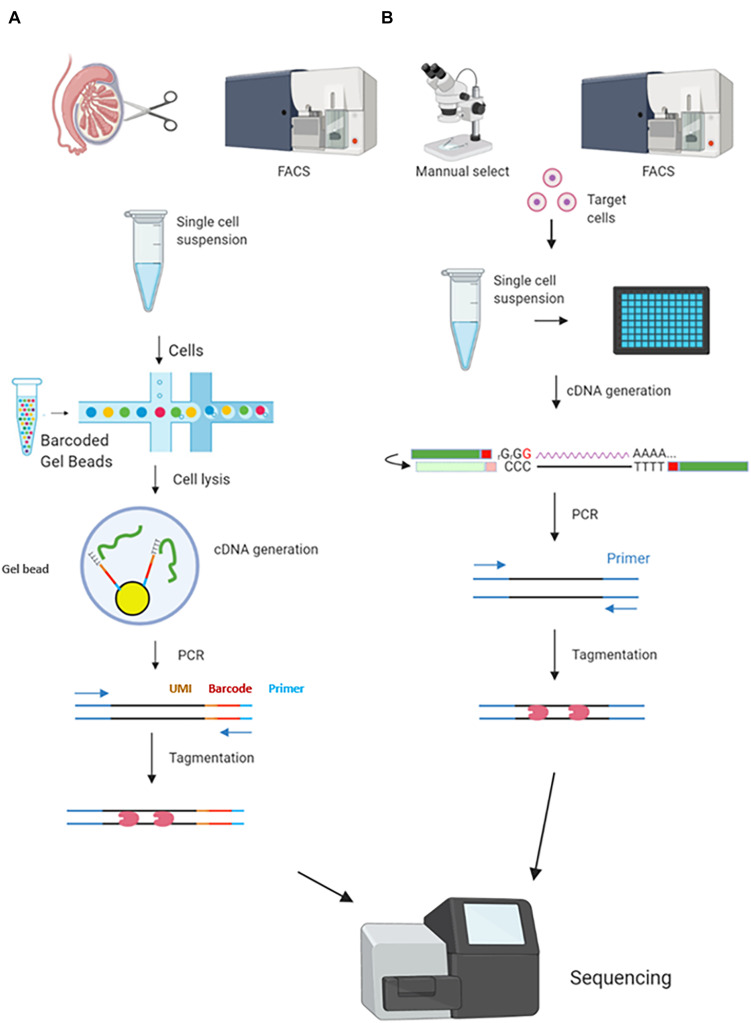
Comparison between two scRNA-seq protocols commonly used in mammalian meiosis studies. Smart-seq2 and Drop-seq are the two most widely used methods in meiosis-related scRNA-seq studies. **(A)** Drop-seq allows a large sample size. A single-cell suspension is generated from testes or ovaries with or without cell sorting and enrichment, which depends on different research purposes. A single cell and a barcoded bead are encapsulated in each water-in-oil droplet. Cells are lysed to release mRNAs in the droplet and the mRNAs were captured by the bead with unique UMIs and barcodes. cDNAs are generated by reverse transcription and the templates were amplified, followed by library preparation. **(B)** Smart-seq2 starts with a small cell sample size. Cells can be collected by manual selection or FACS. Each individual cell is separated into different microtiter plate wells or microfluidics, cDNA will be generated for PCR and then be tagged for sequencing. The cDNA generation and amplification are similar to Drop-seq.

Two well-developed commercial approaches, Drop-seq and Smart-seq2, are the two most widely used methods in meiotic studies. They represent high-throughput 3′ end capture sequencing and full-length sequencing, respectively. Their differences were shown in Table 1. In the Drop-seq method, each individual cell is separated and lysed in an oil droplet with a unique bead carrying barcode sequences including UMI. Those sequences will capture the poly-A ends of mRNAs (3′) to add an “ID” to each mRNA molecule. By doing this, the mRNAs labeled by UMI will avoid amplification noise (Kivioja et al., 2012; Macosko et al., 2015; Ziegenhain et al., 2017; Figure 1A). The most attractive advantage of Drop-seq is its ability to generate a large library from a mixed cell suspension, such as a single cell suspension from entire testes with large cell numbers and multiple cell types. Smart-seq2 has some unique characteristics compared with Drop-seq. First, instead of UMI incorporation and 3′ end mRNA capture, Smart-seq2, similar to Smart-seq, generate full-length mRNA libraries by performing a “Switching Mechanism at 5′ End of RNA Template” workflow (Picelli et al., 2013). Briefly, Smart-seq2 utilizes a reverse transcriptase enzyme to add cytosine residues to cDNAs during mRNA reverse transcription. The enzyme then switches template to RNA, adding guanine residues to 5′ ends. In this way, both 3′ ends and 5′ ends are able to be captured, leading to a full-length coverage (Goetz and Trimarchi, 2012; Figure 1A). Second, to enable individual cell separation, the library preparation of Smart-seq2 needs to be performed on the microfluidic chips or on a multi-well plate (Xin et al., 2016). Smart-seq2 starts with sorted cells, commonly generated by FACS or micromanipulation. Third, the full-length sequencing of Smart-seq2 decreases the mismatch rate for mRNA capture, enabling high sensitivity and accuracy. Thanks to the high sequencing depth, Smart-seq2 can normally capture twice as many gene numbers per cell as Drop-seq does in the same conditions. Smart-seq2 was also proved to maintain the full sequence reads mapping against the 3′ end bias (Ziegenhain et al., 2017). Therefore, Smart-seq2 is able to capture gene isoforms by SNPs. However, Smart-seq2 requires individual cell lysis (cells are commonly separated by wells). Thus, the sample size is usually limited and the library construction cost per cell is relatively high (Table 1 and Figure 1B; Picelli et al., 2013; Ziegenhain et al., 2017; Baran-Gale et al., 2018).

**TABLE 1:** Comparison between Smart-Seq2 and Drop-seq in meiosis studies.

Sequencing method	Smart-Seq2	Drop-seq
UMIs incorporation	NO	YES
Sequencing pattern	Full-length	3′-end capture
Sensitivity (sequencing depth)	High	Low
Able to detect gene isoforms	YES	NO
Targeting cell sample size	Low (96–384 when using plates)	High (100–10,000)
Average library cost (per cell)	11$	0.01$
Special equipment required for library preparation	NO	YES
Common application in meiotic studies	Oocytes	Testis cells

Most male-meiotic scRNA-seq studies utilize the Drop-seq method; in contrast, most female-meiotic scRNA-seq studies choose Smart-seq/Smart-seq2. Their research purposes rather than sample types determine which method(s) to be used. Drop-seq generates 3′ end bias for mapping reads and has lower sequencing coverage compared to Smart-seq2. Therefore, Drop-seq is not suitable to study transcriptional profiles of genes with low abundance. However, this high throughput method allows a large sample size at once, ensuring comparison between numerous cells in the downstream analysis. Therefore, Drop-seq can be suitable for identifying rare cell types, plotting stage-specific transcriptomes, and constructing cell progression trajectories in meiosis studies. These research goals can also be achieved by Drop-seq even in oocyte studies (Niu and Spradling, 2020; Zhao et al., 2020b). In contrast, Smart-seq2 applications in meiotic studies rely on known cell characteristics for efficient cell sorting. Limited sample input and high sensitivity and accuracy are also different from other scRNA-seq methods. These features limit its application within transcriptomic analysis of the known cell types, e.g., comparing transcriptomes between GV and MII oocytes, or comparing abnormal oocytes with normal oocytes at the MII stage (Zhang et al., 2018; Ferrero et al., 2019; Ye et al., 2020; Yang et al., 2021).

### ScRNA-Seq Downstream Analysis for Meiosis Studies

**FIGURE 2:**
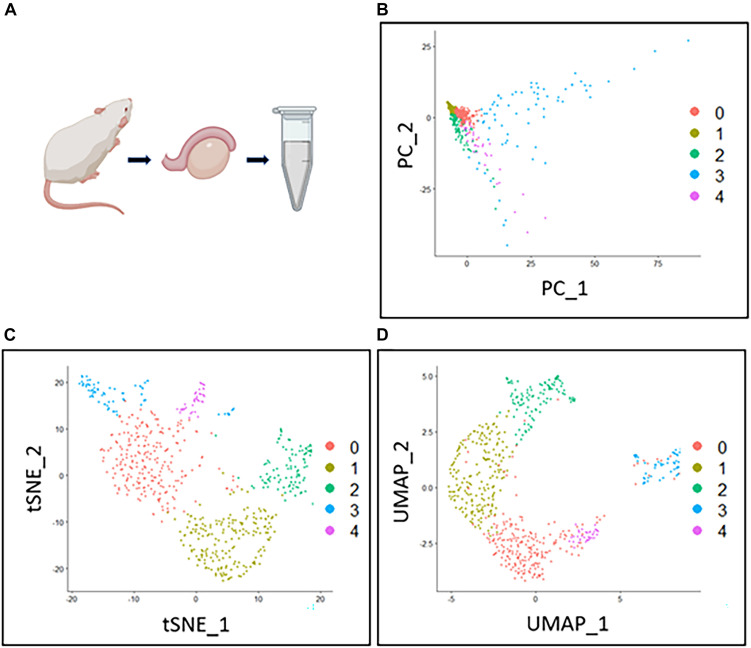
Comparison between three dimension-reduction methods of scRNA-seq data analysis. The Drop-seq testis cell data was progressed by quality control and normalization. The polished data then underwent three different dimension-reduction methods for cell clustering. Each dot in **(B–D)** represents a cell. **(A)** The mouse testis data is from a previous publication (Jung et al., 2019). **(B)** The filtered testis data were processed by PCA and plotted by the first two principal components. Cell clusters were generated and shown in different colors. **(C)** The same dataset is processed by TSNE for clustering and plotted by the first two dimensions. Cluster separation of TSNE is better compared to other methods. **(D)** The same dataset is processed by UMAP for clustering and plotted by the first two dimensions. The distance between different clusters reflects the farther or closer relationships between the cell types.

**FIGURE 3:**
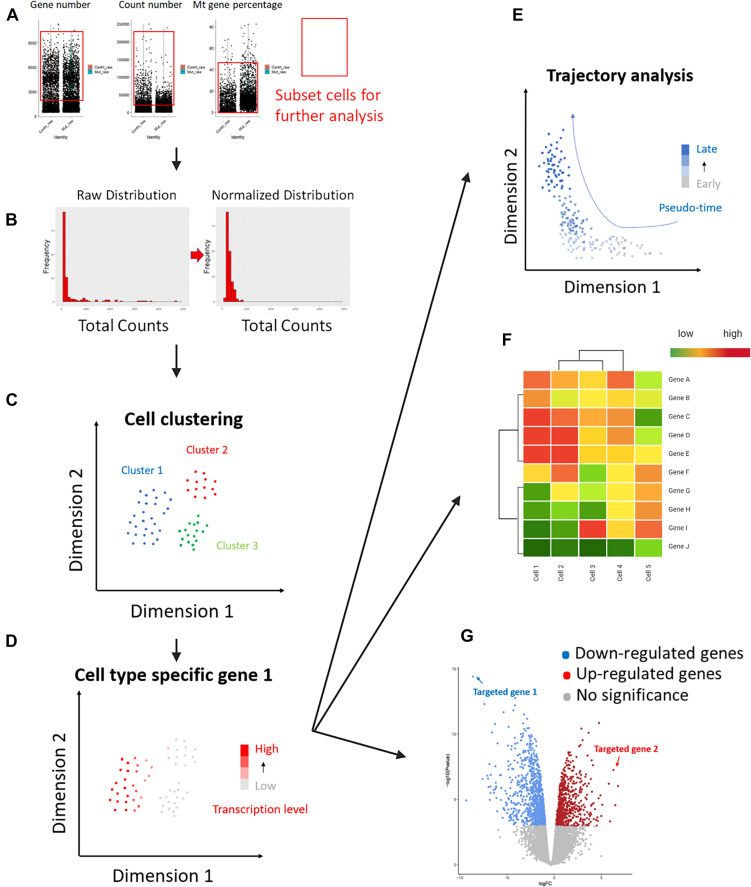
Downstream analysis workflow of commonly used scRNA-seq. Key steps of the scRNA-seq downstream analysis were summarized here (The figure is created with BioRender.com). **(A)** Quality control evaluated cell quality and filtered qualified cells by captured gene numbers, mRNA total counts, and mitochondrial mRNA percentages. Red rectangles highlighted the cells selected according to the three parameters, respectively. The rest cells were considered as “low quality “cells and not used for the following analysis steps. **(B)** The normalization method was applied to remove technique deviation, especially different sequencing depth between each individual cell. The mRNA-total-count distribution from the normalized cells (right graph) is closer to normal distribution compared to the raw counts (left graph). **(C)** Different dimension-reduction methods can be used for cell clustering (see Figure 2 for details). Cells were separated by their transcriptional features. **(D)** Cell type identification. Separated cell clusters can be identified by using known cell-type-specific markers. **(E)** Alternative downstream analysis can be applied for different research purposes. Pseudo-time trajectory analysis can be used for tracking cell progression from early to late stages. In meiosis-related studies, trajectory analysis is commonly used for tracking spermatogenesis. **(F)** Cell-expression data can be used to generate cell clustering heatmaps. Similar cells will be clustered together on the heatmap, and the gene expression data showing their homology and heterogeneity will be shown. In this way, potential gene markers for certain cell types can be easily determined. **(G)** Differential Gene Expression (DGE) analysis compares differential gene transcription levels between two cell groups or datasets. The identified up- and down-regulated genes provide important information for diagnosis and treatment.

The following downstream analysis of scRNA-seq is also essential for performing scRNA-seq (Figures 3A–G). Several downstream analysis approaches can be used to identify cell types accurately. As meiosis contains a lot of substages, some of which are hard to distinguish by cytological features like sizes and shapes, a transcriptome-based cell identification is important for cell identification. Since cells are featured by thousands of genes, current single-cell clustering approaches depend on dimension-reduction methods, such as Principal Component Analysis (PCA), Uniform Manifold Approximation and Projection (UMAP), and T-distributed Stochastic Neighbor Embedding (t-SNE) (Figures 2B–D; Pearson, 1901; Van Der Maaten and Hinton, 2008; McInnes et al., 2018). For a transcriptome matrix having multiple cells and numerous genes, dimension-reduction analyses transfer data to low-dimension states and preserve basic heterogenetic information. The difference between those methods relies on different algorithms that are used to calculate the distances when performing cell clustering. Specifically, t-SNE and UMAP use non-linear graph-based dimension reduction algorithms to define the distance among cells. The adjusted clustering strategies provide better visualization for cell-group identifications. It is widely accepted that tSNE can more efficiently provide information on cluster separation than PCA; while UMAP can better show the relationship between cell clusters (Figures 2A–D; Jung et al., 2019). The application of the aforementioned methods simplifies the cell transcriptional features from thousands of genes to a limited number of principal dimensions. If the first two principal dimensions are taken, cells with similar transcriptional features can be clustered together shown on a two-dimensional plot (Suzuki et al., 2019).

How can we know which stage these clusters represent? Using stage-specific marker genes to identify cell types is a commonly used staging method for scRNA-seq analysis. Some genes only express at certain meiotic stages, the high expression of those genes can be used as an indicator to track the target cell types. For instance, genes encoding transition proteins (TNP1 and TNP2) are useful ES markers because they only express in ES to replace histones (Meistrich et al., 2003). The function that links known markers to cell clusters has been integrated into analysis software, such as the Seurat package for R (Butler et al., 2018; Do et al., 2018; Stuart et al., 2019). However, high turnover rates of mRNA and limited marker numbers still challenge the identification of specific germ-cell substages.

Trajectory-based differential expression analysis for scRNA-seq data is a novel tool for discovering hidden sub-transition stages during spermatogenesis. Like other biological progressions, germline cells undergo gradual transcriptional changes along the progression of meiosis. This gradual transcriptome transition can be reflected as continuous cell clusters in low-dimension plots by single-cell trajectory analysis (Trapnell et al., 2014). Pseudotime analysis provides an efficient way to obtain more continuous cell-cluster trajectories, mimicking real kinetics of germ-cell development (Figure 3E; Campbell and Yau, 2018). This method has been used to identify the renewal and differentiation initiation of spermatogonia stem cells (SSC) by using marker genes (Hermann et al., 2018). As a commonly used pseudotime analysis tool that is independent of known markers, Monocle (a Bioconductor package) orders cells into trajectory trees and branches by calculating transcriptional relations (Trapnell et al., 2014). Labeling a trajectory tree with known markers can be used to define the transitional cell stages of the known stages without specific markers, e.g., an unknown stage in the middle of two continuously progressed cell stages along the trajectory can be recognized as a transition stage between the two known stages.

### Adding More “Markers” in Our Toolbox: Cell-Type Marker Genes for Identifying Specific Meiotic Substages in scRNA-Seq Studies

As mentioned before, meiotic marker genes are important information used in scRNA-seq downstream analysis. Researchers not only used widely accepted meiotic marker genes for cell-type identification in single-cell data sets, but also analyzed differentiated gene expression among different cell clusters to reversely identify cell-type-specific or stage-specific meiotic marker genes (Figure 3E). Taking the advantages of DGE analysis, marker genes are selected by comparing the genomic transcription levels between different cell groups and those top genes in each group represent the most unique transcriptions. Most of the previous studies staged germline cells via cytological approaches. Due to the non-synchronized pattern of many meiosis-related genes, some of the protein-coding genes may transcribe early but translate late. Therefore, previous genes used for meiotic cell identification may not be compatible for scRNA-seq data. ScRNA-seq-specific marker genes were needed. Here, we summarized those markers from single-cell data that can be used for identifying spermatocytes and oocytes at different meiotic stages, we also collected data to compare marker genes between male and female (Table 2; Guo et al., 2017, 2018; Li et al., 2017; Fayomi and Orwig, 2018; Green et al., 2018; Hermann et al., 2018; Lukassen et al., 2018; Ernst et al., 2019; Tan and Wilkinson, 2019; Ge et al., 2020; Niu and Spradling, 2020; Wang et al., 2020; Zhao et al., 2020a).

**TABLE 2:** Meiotic gene markers for prophase I in spermatogenesis and oogenesis.

Stage/Marker identity	Human markers (male)	Mouse markers (male)	Mouse markers (male Specific)	Mouse markers (female)	Mouse markers (female specific)
Leptotene	Scml1, Smc1b, Herc5, Zcwpw1	Mdk, Ccnb3, H2bfm, Scml1	Rhox2h, Fthl17-ps3, Rhox2d, Mageb18, A830018L16Rik, Rhox2a, Dppa5a, Gml, Gm364, Rhox2h, Fthl17-ps3, Rhox2d, Mageb18, A830018L16Rik, Rhox2a, Dppa5a, Gml, Gm364, Ccnb3,	Actb, Hmgb1, Rpl6, Hmgb2, Rpl39, Stra8, Dazl, Smc1b, Hells, Sycp1, Sycp3, Zcwpw1, Tex30, Tex101, Rec8, Tuba3a, Cited1, Syce1, Cdkn2a	Actb, Rpl24, Ptma, Tubb5, Eef1g, Rsp3
Zygotene	Tpte, Sod1, Loc100507384, Linc00668, Tdrg1	Sycp1, Sycp3, Prss50, Tex101			M1ap, stra8, Tuba3a, Selenok, Snu13, Haus8, Med21
Pachytene	Ccdc112, C9orf57, Piwil1, Prok2, Adam2, Mgat4d	Piwil1, Tmem30c, Mllt10, Rsph1, Cdc42ep3	Rsph1, Lyar, Calm1, Ldhc, Atxn7l3b, Rbakdn, Pabpc6, Gkap1, Cox8c	Tsga10, Ankrd31, Grk4, Ndufa1, Eif4a2, 4930447C04Rik, Malat1, H1f0, Uba52, Calr	Hist1h2aa, Rad51ap2, Zhx1, Zmym6, mt-Nd6, Hist1h4d
Diplotene	Gyg1, Aurka, Zc2hc1c, Ccnb2, Tmigd3, Spata16	Pou5f2, Mxra8, Ggn, Wdr20rt, Rassf1	Pou5f2, Mxra8, 4932702P03Rik, Ggn, Gm8879, 1700108N11Rik, Wdr20rt, 4930515G01Rik, Rassf1, 4933402N22Rik	Nmnat3	Gm13269, Gm27164, Gm44601, Gm49368, Grid2, Syce3, Olfr678, Ablim1, Uba52, Pet2, Brd2

*This table summarized markers for four substages in meiosis prophase I. Human and mouse markers were listed. Those markers come from different resources (see citations in the text). Markers in the table were identified from the total 13 publications. Specifically, the meiotic markers for male are generated by comparing differentiated genes at same or similar stage from different scRNA-seq datasets, all the marker genes selected were identified as markers by DGE analysis from at least 2 different studies to ensure the consistency. For mouse female meiotic markers, all the data comes from Niu and Spradling (2020) because this study is the only high throughput Drop-seq study that dissect transcriptomic features of oocytes in a fine scale. We also create male and female specific marker list by comparing Niu’s study to Drop-seq based male studies that targeted at similar meiotic stages. Genes that were identified as significant differentiated-expressed genes (p < 0.05) at certain stage in either male or female were put into the list.*

## Conclusion and Future Directions

ScRNA-seq is a high-throughput sequencing method that is widely used in research. It allows researchers to study transcriptomes at the single-cell level. Different scRNA-seq technologies have been developed for various research requirements and purposes. Those technologies strengthened the researcher’s ability to study meiosis, especially in mammals. Conventional bulk RNA-seq combined with cell synchronization and sorting has limitations in studying minor cell groups and specialized cell substages. In contrast, scRNA-seq takes advantage of dimension-reduction methods for cell clustering and allows accurate cell identification. In mammalian meiosis studies, current clustering methods can distinguish between each substage throughout meiotic prophase I. Many downstream-analysis methods have been developed to identify new cell types and progression tracks. For example, pseudo-time trajectory analysis has already been used in meiosis progression studies.

ScRNA-seq is also used more frequently as a diagnostic tool for meiosis-related diseases. Since the causes of many meiosis-related diseases remain unknown, scRNA-seq of germ cells from patients and healthy donors can be used to investigate potential mechanisms of these diseases by analyzing their differential gene expression.

Meanwhile, there are still some challenges of scRNA-seq that limit its broader application in meiotic-related fields. First, current scRNA-seq, especially NGS-based platforms, induces great RNA loss, leading to low sequencing depth (Chen et al., 2019). For example, NGS-based sequencing typically reaches as low as 10 versus. 40% for full-length sequencing depth (Tirosh et al., 2016; Yuan et al., 2017; Nguyen et al., 2018). Low sequencing depth can cause more background or noise than bulk RNA-seq, making it difficult to capture the RNAs with low abundance, like lncRNAs. The number of genes that can be captured from scRNA-seq is also normally lower than bulk RNA-seq (Saliba et al., 2014; Haque et al., 2017). Second, the NGS-based sequencing approaches typically only capture the 3′-end of each mRNA and break mRNA into small pieces. Thus, this approach often fails to maintain the full sequence of the RNAs and cause information loss. Another sequencing information loss is because it is hard to detect RNA isoform variants, RNA modifications, and short length RNAs (such as microRNAs) (Macosko et al., 2015; Heath et al., 2016). It is important to capture microRNAs in meiosis-related studies because microRNAs play important roles in mammalian meiosis (Walker, 2021). This limitation produces a contradiction between high-throughput and high-sensitive scRNA-seq approaches. While full length scRNA-seq, e.g., Smart-seq2, partially solves the aforementioned problems. However, limited cell numbers can be sequenced at a time via Smart-seq2, which cannot meet the requirement for cell heterogeneity and progression studies of meiosis, especially spermatogenesis (Picelli et al., 2013; Ziegenhain et al., 2017). Third, current scRNA-seq approaches normally result in variant sequencing depth in each individual cell, creating challenges for downstream normalization analysis to achieve real biological features (Bacher et al., 2017; Rizzetto et al., 2017; Hafemeister and Satija, 2019). Overall, the limitations for scRNA-seq approaches still need to be overcome, especially for future meiotic studies.

For meiotic studies, the first future direction for scRNA-seq technology would be increasing sensitivity and accuracy for high-throughput library preparing protocols. The improvement for higher sequencing depth, lower technical noise, and the ability to capture more types of mRNA will help decipher deeper molecular mechanisms for meiosis. For instance, important meiotic genes with low transcription counts can be further studied, the accurate transcriptomic identification will also lead to new insight into transcriptional regulations during meiosis. Information about microRNA from improved scRNA-seq will broaden our knowledge on meiosis.

The second future direction for meiotic scRNA-seq studies will be the new technologies combining with and/or based on scRNA-seq. ScRNA-seq can bind other NGS methods to incorporate single-cell transcriptomic with genomic, proteomic, and epigenetic information, which together were named “single cell multi-omics technology”(Hu et al., 2018). Currently, the multi-omics technologies are experiencing an explosive growth as multiple protocols have been developed continuously, e.g., scG&T-seq, scMT-seq, scGESTALT, and ECCITE-seq (Macaulay et al., 2015; Angermueller et al., 2016; Raj et al., 2018; Mimitou et al., 2019). We expect to see more single-cell sequencing methods can be integrated together to answer the challenging questions in meiosis, e.g., how different factors work together to generate heterogeneous regulations in meiotic cells.

Finally, besides the improvement in library preparation and sequencing technologies, more advanced and mature computational pipelines can help to dig up the increasing scRNA-seq datasets. First, overcoming batch effects between different scRNA-seq experiments and platforms can potentially integrate analysis across multiple scRNA-seq datasets. The newly developed algorithms have shed light on mining the existing data (reviewed by Forcato et al., 2021). Second, the variable analysis approaches increase the difficulties of evaluating different scRNA-seq studies. With the development of bioinformatic tools, the appearance of “golden standard pipelines” will normalize the interpretation of scRNA-seq data, thus, generate more comprehensive transcriptional references for meiotic studies.

## Author Contributions

YP and HQ contributed to writing and editing of this review. Both authors contributed to the article and approved the submitted version.

## Conflict of Interest

The authors declare that the research was conducted in the absence of any commercial or financial relationships that could be construed as a potential conflict of interest.

## Publisher’s Note

All claims expressed in this article are solely those of the authors and do not necessarily represent those of their affiliated organizations, or those of the publisher, the editors and the reviewers. Any product that may be evaluated in this article, or claim that may be made by its manufacturer, is not guaranteed or endorsed by the publisher.
